# Growth Dynamics of Bacterial Populations in a Two-Compartment Biofilm Bioreactor Designed for Continuous Surfactin Biosynthesis

**DOI:** 10.3390/microorganisms8050679

**Published:** 2020-05-07

**Authors:** Hannah Luise Brück, François Coutte, Pascal Dhulster, Sébastien Gofflot, Philippe Jacques, Frank Delvigne

**Affiliations:** 1 MiPI, TERRA Teaching and Research Centre, Joint Research Unit BioEcoAgro N° 1158, Gembloux Agro-Bio Tech, University Liège, University Lille, INRAE, UPJV, YNCREA, University Artois, University Littoral Côte d’Opale, B-5030 Gembloux, Belgium; hannah.bruck@doct.uliege.be (H.L.B.); philippe.jacques@uliege.be (P.J.); 2 ICV—Institut Charles Viollette, Joint Research Unit BioEcoAgro N° 1158, University Lille, INRAE, University Liège, UPJV, YNCREA, University Artois, University Littoral Côte d’Opale, F-59000 Lille, France; francois.coutte@polytech-lille.fr (F.C.); pascal.dhulster@univ-lille.fr (P.D.); 3 Walloon Agricultural Research Center (CRA-W), Agricultural Product Technology Unit, Chaussée de Namur, 24, B-5030 Gembloux, Belgium; s.gofflot@cra.wallonie.be

**Keywords:** biofilm reactor, continuous bioprocessing, biosurfactants, *B. subtilis*, exopolysaccharides

## Abstract

Biofilm bioreactors are promising systems for continuous biosurfactant production since they provide process stability through cell immobilization and avoid foam formation. In this work, a two-compartment biofilm bioreactor was designed consisting of a stirred tank reactor and a trickle-bed reactor containing a structured metal packing for biofilm formation. A strong and poor biofilm forming *B. subtilis* 168 strain due to restored exopolysaccharides (EPS) production or not were cultivated in the system to study the growth behavior of the planktonic and biofilm population for the establishment of a growth model. A high dilution rate was used in order to promote biofilm formation on the packing and wash out unwanted planktonic cells. Biofilm development kinetics on the packing were assessed through a total organic carbon mass balance. The EPS^+^ strain showed a significantly improved performance in terms of adhesion capacity and surfactin production. The mean surfactin productivity of the EPS^+^ strain was about 37% higher during the continuous cultivation compared to the EPS^-^ strain. The substrate consumption together with the planktonic cell and biofilm development were properly predicted by the model (α = 0.05). The results show the efficiency of the biofilm bioreactor for continuous surfactin production using an EPS producing strain.

## 1. Introduction

Most of the biotechnological processes are based on planktonic cells in suspension in the cultivation medium [1]. Bioreactor operations are often limited to batch and fed-batch processes, although continuous processing would be more cost-efficient due to reduced downtime for the reactor cleaning, preparation and cell growth [1]. Cell retention and a long-term cell viability represent the main challenges in a continuous reactor [1].

Natural cell immobilization through biofilm formation presents an interesting alternative technique to design new continuous bioprocesses. In nature, biofilms are the predominant lifestyle of bacteria. A biofilm is a multicellular community of one or several bacterial species that is protected through a self-produced polymer matrix. Thereby, biofilms possess an enhanced tolerance to toxic substrates or products compared to the cells in planktonic state and thus, remain viable under unfavorable conditions as well as are able to regenerate themselves [2,3]. Due to the high biomass density in biofilms and their stability, biofilm reactors have a high potential for long-term fermentation processes [4,5]. However, the biofilm community is highly heterogeneous due to cell differentiation as a result of adaption to nutrients and oxygen gradients inside the biofilm. This heterogeneity makes it challenging to control the growth of the biofilm in the bioreactor.

Many microorganisms are able to grow naturally on diverse surfaces [1,3]. In the medical sector, harmful biofilms are a heavy burden since they provoke severe infections and have detrimental effects on human health [5]. In industrial installation, biofilms can be responsible for biofouling and contaminations and thus present high hygienic risks [6,7]. Yet, many industrial applications exist that are taking advantage of biofilms by using them as workhorses. These beneficial biofilms are for example used in the waste-water treatment, bioremediation or the production of bioenergy [8,9,10].

*Bacillus* spp. are well known for their ability to produce different families of biosurfactant lipopeptides with high application potential such as surfactins, fengycins and iturins [11]. Previous works have shown that cell immobilization in biofilm bioreactors is particularly favorable for the production of the above-mentioned compounds and allows the design of bioprocesses avoiding excessive foam formation [12,13,14,15,16], although biofilm development is a highly dynamic process with instabilities depending on the environmental conditions, such as the release of cells back into the liquid phase upon biofilm disruption. Biofilm development is difficult to assess during the cultivation due to restricted access to the support where the biofilm is growing. It is thus important to develop new measurement and control strategies for monitoring biofilm development and for designing robust processes.

In environmental biotechnology, mathematical modelling of biofilms is used to plan, design, optimize and evaluate processes in wastewater treatment plants [17]. The implementation of biofilm models permits to calculate the development over time of microbial species and substrates [18] and to get insights into relevant parameters that control the performance of the biofilm process [19]. It is important to select only the most relevant parameter to describe the physiological state of the organism and the behavior of the system to reduce the complexity of the model [20]. These models are developed through the set-up of mass balance equations for the relevant components involved in the bioprocess and the description of the corresponding kinetics expressions [20]. The components can generally be divided into two categories: the microorganisms and the consumed or produced materials of the microorganisms [19]. However, the mathematical modeling of biofilm reactors is not always straight forward due to the complexity of biological reactions involved in substrate conversion and the lack of accurate kinetic parameters for the biofilm development [21]. The approach of inverse modeling has been shown to be an attractive method for the numerical evaluation of kinetic parameters in biofilm processes. Through the validation of the biofilm model with the measured data, the parameters are determined in the way that the observed process behavior is approximately represented through the model [21].

In this work, a lab-scale two-compartment microbial system composed of a trickle-bed biofilm bioreactor and a stirred tank reactor was designed for the production of surfactin. Through a continuous operation mode, a strong selective pressure was induced on the cell populations. In the actual bioreactor design, biofilm development is promoted to achieve a high cell density on the packing element to increase the production yield. The planktonic cells, in contrast, are not favored and eliminated through a high dilution rate in order to simplify the downstream process of the secreted product. Experimental data are collected with a strong- and poor-biofilm-forming strain derived from *B. subtilis* 168 for establishing a growth model in order to get a deeper insight into the populations’ behavior. The model is especially useful for predicting the kinetics of the biofilm development on the packing elements, a parameter difficult to assess during cultivation. Moreover, additional information on the system behavior can be obtained through the processing of the model. This provides important information for further process improvement through strain engineering.

## 2. Materials and Methods 

### 2.1. Strains

The two *B. subtilis* strains used in this study and their corresponding genotype are listed in Table 1. Both strains were derived from the laboratory strain *B. subtilis* 168 (*trpC2*, *sfp*^0^, *epsC*^0^).

### 2.2. Biofilm Growth Visualization on Drip-Flow Reactor Coupons

The two *B. subtilis* strains were cultivated in a drip-flow reactor device during 48 h on silicone coupons, exactly as described in [24]. The biofilm is developing on the surface of the coupons which permits to observe easily different biofilm phenotypes. The biofilm images were taken with a Samsung Dual Pixel 12 MP camera at the end of cultivation.

### 2.3. Design of the Lab-Scale Trickle-Bed Biofilm Reactor and Culture Conditions

**Figure 1:**
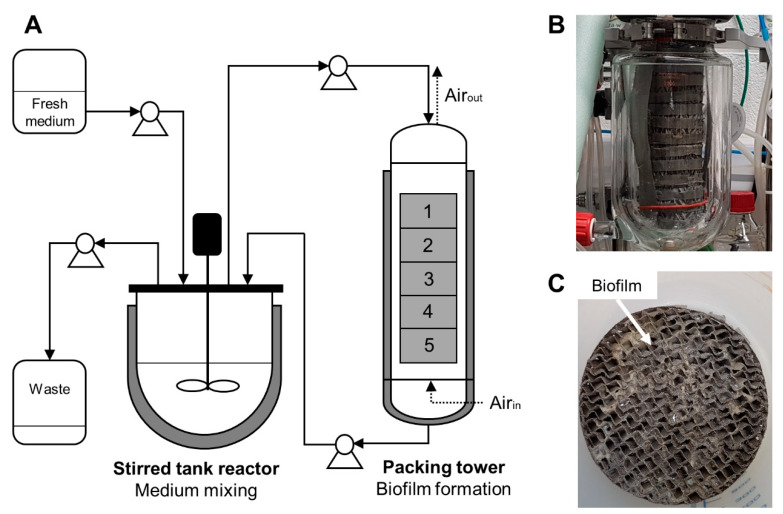
(**A**) Experimental set-up of the lab-scale trickle-bed biofilm reactor. (**B**) Packing tower (side view). (**C**) Top view image of one stainless steel structured packing element colonized by a biofilm.

A lab-scale (2 L) trickle-bed biofilm bioreactor has been designed on the basis of previous works carried out on a 20 L bioreactor containing a structured stainless steel packing element [16,25]. The experimental set-up of the designed reactor is presented in Figure 1.

For the lab-scale trickle-bed biofilm bioreactor, the system was separated into two main reactors: one for medium mixing and another that contained a tower of five structured metal packing elements for biofilm formation. The packing elements are composed of assembled corrugated gauze stainless steel sheets, a hydrophobic material with good wettability capacities (Laboratory packings, 83 × 55 mm, Sulzer Chemtech, Winterthur, Switzerland). Moreover, the metal structured packing provides an increased gas/liquid mass transfer.

The medium was recirculated continuously between these two devices with a flow rate of 85 mL min^−1^. The medium was mixed at 300 rpm in the reactor. The mixing reactor was a conventional 2 L bioreactor (BIOSTAT B Plus, Sartorius Stedim, Schaerbeek, Belgium) whereas the reactor containing the packing elements was composed of a previous 2 L chemical reactor with a double jacket for temperature regulation (Reactor-Ready, Radleys, Shire Hill, Saffron Walden (Essex), UK). Since this type of reactor does not possess a condenser which is crucial to avoid filter clogging and pressure problems due to medium evaporation, the gas outlet was refrigerated by an additional cooling system to reduce evaporation in the packing reactor. The temperature of both reactors was regulated to 37 °C. For security, the gas outlet was connected to a reservoir bottle with filters in case of too strong evaporation to collect the condensate. During the cultivation, there is no aeration in the mixing reactor. Air (1 L min^−1^) is injected only on the downside of the packing reactor to prevent foam formation. The medium is injected on the upper side of the packing reactor and then flows down by gravity on the packing elements. Oxygen mass transfer is promoted through the counter-current flow of the injected air and the liquid. For the continuous process mode, an entry to and exit from the mixing reactor was added. Samples were taken from the mixing reactor by means of a sterile syringe.

For the reactor inoculation, a series of pre-cultures was prepared. First, 2 mL of lysogeny broth (LB) medium (10 g L^−1^ tryptone, 5 g L^−1^ yeast extract, 10 g L^−1^ NaCl) was inoculated with a colony. The first pre-culture was incubated for about 6 h at 37 °C and 160 rpm. Then, a second pre-culture was prepared by a 10 times dilution of pre-culture I in LB medium. The second pre-culture was incubated overnight at 37 °C and 160 rpm and then 10 times diluted with Landy MOPS medium (20 g L^−1^ glucose, 5 g L^−1^ glutamic acid, 1 g L^−1^ yeast extract, 0.5 g L^−1^ MgSO_4_, 1 g L^−1^ K_2_HPO_4_, 0.5 g L^−1^ KCl, 1.6 mg L^−1^ CuSO_4_, 1.2 mg L^−1^ MnSO_4_, 0.4 mg L^−1^ FeSO_4_, 21 g L^−1^ MOPS, 1.6 mg L^−1^ tryptophan) to prepare the main pre-culture. The main pre-culture was grown to an OD_600 nm_ between 2 and 3 and then used to inoculate the reactor (1 L working volume) with an OD_600 nm_ of 0.2 (corresponds to ~0.08 g L^−1^ cell dry weight). Before inoculation, the cells were washed once in a 0.9% NaCl solution to synchronize the cells and eliminate the produced primary and secondary metabolites. For this purpose, the cell culture was centrifuged (10 min at 2700× *g*) and the supernatant was discarded. The remaining cell pellet was resuspended in a 0.9% NaCl solution and then used to inoculate the reactor. The reactor contained Landy medium without MOPS buffer. The reactor pH regulation was executed using 1 M H_3_PO_4_ as acid and 3 M NaOH as base. The pH in the reactor was set at 7.0. To the reactor medium 50 µL L^−1^ of a silicone-free organic antifoaming agent (TEGO^®^ Antifoam KS911, Evonik, Essen, Germany) was added. The culture was started with a batch fermentation during 16 h to increase the cell number in the reactor and to promote cell adhesion and biofilm development on the support. Then, the continuous phase was launched during ~28 h with a dilution rate of D = 0.5 h^−1^ which corresponds to a feeding rate of 500 mL h^−1^. Two replicates of the biofilm cultivation experiments were performed per strain.

### 2.4. Determination of the Mean Residence Time in the Packing Tower 

For the mean residence time determination in the packing tower, tracer particles (1 µm) were injected on the top of the packing tower with a flow rate of 85 mL min^−1^ and collected at the packing tower exit at time intervals of 5 s. The collected particles were counted by flow cytometry (BF Accuri^TM^ C6, BD Biosciences, Erembodegem-Dorp, Belgium). The mean residence time was then calculated with the measured tracer concentration over the time by Equation (1):

(1) $$\bar{t}=\frac{\int_{0}^{\infty }t*c(t)dt}{\int_{0}^{\infty }c(t)dt}$$

where c represents the measured tracer concentration at time point t.

### 2.5. Biomass Dry Weight Determination

The cell culture samples were centrifuged (10 min at 2400× *g*) and the supernatant was collected to determine the surfactin concentration as described in Section 2.7. The remaining cell pellets were washed by resuspending them in distilled water followed by centrifugation. After the centrifugation, the supernatant was discarded, and the remaining cell pellet was re-dissolved in water and filled into a pre-weighted aluminum cup. The biomass sample in the aluminum cup has been dried in the oven at 105 °C and weighted after 48 h to determine the corresponding cell dry weight.

After the cultivation, the packing elements were left for two hours in the reactor to let drain the residual liquid before they were weighted with the wet biofilm. The corresponding biofilm dry weight was then calculated using a previously determined biofilm dry weight percentage (see Data S1).

### 2.6. Glucose Analysis

Glucose concentration was analyzed in the supernatant using high-performance liquid chromatography (HPLC). A Waters Acquity UPLC^®^ H-Class System (Waters, Zellik, Belgium) with an ion-exchange Aminex HPX-87H column 7.8 × 300 mm (Bio-Rad Laboratories N.V., Temse, Belgium) heated up to 50 °C was used for analysis. A metabolite analysis was carried out with an isocratic flow rate of 0.6 mL min^−1^ for 25 min. The mobile phase was composed of water containing 5 mM H_2_SO_4_. Elution profiles were monitored through a Waters Acquity^®^ Refractive Index Detector (RID) (Waters, Zellik, Belgium). A glucose standard solution (Sigma-Aldrich, Overijse, Belgium) was used to determine the retention time and to establish a calibration curve.

### 2.7. Surfactin Analysis

The supernatants from the centrifuged cell culture samples were filtered (0.2 µm) and the surfactin concentration was determined by reversed-phase HPLC (Agilent 1100 Series HPLC Value System, Agilent Technologies, Diegem, Belgium) with an Eclipse XDB C−18 column (3.5 µm, 2.1 × 150 mm) (Agilent Technologies, Diegem, Belgium). The HPLC analysis method was based on an isocratic elution profile with a mobile phase composition of 80% acetonitrile and 20% water containing 0.1% trifluoroacetic acid (TFA). The flow rate was set at 0.4 mL min^−1^ with an analysis time of 22 min per sample. The surfactin molecules were detected by UV at 214 nm. Purified surfactin samples (>98%) (Lipofabrik, Villeneuve d’Ascq, France) were injected to identify the retention time of the surfactin molecules and to determine a calibration curve.

### 2.8. Total Organic Carbon Analysis and Establishment of the Mass Balance

Total organic carbon (TOC) measurements of the culture medium were performed in order to estimate the TOC consumption of the cells. The planktonic cells were separated from the bulk medium by centrifugation. Subsequently, the TOC content of the culture medium was measured using a Lotix Combustion TOC Analyzer (TELEDYNE TEKMAR, Mason, OH, USA). The diluted culture medium samples were injected into the combustion tube where the samples were completely oxidized to CO_2_ through catalytic combustion at 720 °C. Subsequently, the produced CO_2_ was detected by flow-through non-dispersive infrared spectroscopy. The instrument was calibrated with a standard solution of potassium hydrogen phthalate for a calibration range of 0 to 20 ppm.

A TOC mass balance for the batch and continuous cultivation phase has been established to estimate the TOC consumed by the cells present in the system. The TOC consumption in the batch phase was determined by Equation (2):

(2) $${\mathrm{TOC}}_{\mathrm{consumed},t1}={\mathrm{TOC}}_{\mathrm{medium},t0}-{\mathrm{TOC}}_{\mathrm{medium},t1}$$

and for the continuous phase, with Equation (3):

(3) $${\mathrm{TOC}}_{\mathrm{consumed},t1}={(\mathrm{TOC}}_{\mathrm{in}\mathrm{medium},t0}-{\mathrm{TOC}}_{\mathrm{medium},t1})*D*\Delta t$$

where TOC represents the amount of total organic carbon in g at a certain time point t in h, D is the dilution rate in h^−1^ and Δt the difference between time point t_0_ and t_1_ in h. For the mass balance of the continuous phase, it was assumed that the TOC consumption rate remains constant during the measured time interval.

### 2.9. Biofilm Reactor Compartment Model

The trickle-bed bioreactor system can be simplified into two main compartments representing the main places of residence in the system for sessile and planktonic cells. The first compartment comprises the sessile cells which form a biofilm on the packing tower where cells have unlimited access to dissolved oxygen. In the second compartment, the planktonic cells are growing under limited dissolved oxygen conditions. The overall growth conditions for the cells are better in the packing tower since there is more dissolved oxygen available as well as enough nutrients since the medium is continuously recirculated. In the present system, the contact between the injected gas and the adhered bacteria on the packing elements is strongly enhanced which favors additionally an interfacial oxygen transfer through a direct bacteria-air contact contributing to an increased total oxygen transfer [26].

The objective of this process is to increase the adhered biomass on the packing tower and reduce or eliminate the presence of planktonic cells in the stirred tank reactor through a high dilution rate (D > µ). Increased cell density on the packing elements means increased production yield. The elimination of planktonic cells would strongly facilitate the downstream process since the secreted product in the bulk medium could be easily recovered. This means that the aim for this system is not to reach a steady state as in a normal chemostat reactor (µ = D), but a steady state with a planktonic cells number close to zero and a continuously and stable growing biofilm.

**Figure 2:**
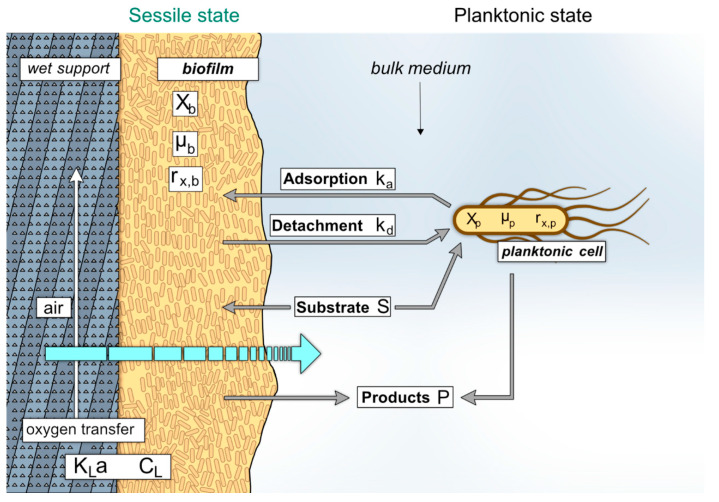
Schematic description of the growth model developed for the two-compartment trickle-bed biofilm reactor. The transition of the cells between the two compartments from the sessile (1) and planktonic (2) state and vice versa takes place in the packing tower (see Figure 1 for a scheme of the cultivation set-up). The scheme shows an enlarged view of a support element inserted in the packing tower and describes the parameters that were used to build the growth model (see Table 2 for a detailed description).

In this work, the development of the two populations (sessile and planktonic cells) was investigated in order to acquire more information about their behavior for further process optimization. By means of experimental data, a simple ordinary differential equations (ODEs) model was established on the basis of bacterial growth equations. A schematic description of the model is presented in Figure 2.

### 2.10. Determination of the Volumetric Oxygen Mass-Transfer Coefficient K_L_a in the Stirred Tank Reactor by Dynamic Gassing-In/Gassing-Out Method

The oxygen transfer rate from a gas to a liquid phase is given by Equation (4):

(4) $${\mathrm{OTR}=K}_{L}{a(C}_{\mathrm{sat}}-C_{L})$$

where K_L_a is the volumetric oxygen mass-transfer coefficient, C_sat_ the oxygen concentration at saturation in the liquid medium in equilibrium to the gas phase and C_L_ the dissolved oxygen concentration in the liquid medium [27]. In a stirred tank reactor where the liquid phase is well mixed, the accumulation of oxygen in the liquid phase can be described through Equation (5):

(5) $$\frac{{\mathrm{dC}}_{L}}{\mathrm{dt}}=\mathrm{OTR}-\mathrm{OUR}$$

where OTR is the oxygen transfer rate from the gas to the liquid and the OUR represents the oxygen uptake rate by the biomass [27]. Since the volumetric oxygen mass-transfer coefficient K_L_a has been measured in the absence of biomass, OUR = 0, Equation (5) can be simplified to Equation (6):

(6) $$\frac{{\mathrm{dC}}_{L}}{\mathrm{dt}}=K_{L}{a(C}_{\mathrm{sat}}-C_{L}).$$

And thus can be transformed into Equation (7):

(7) $$\ln(\frac{C_{\mathrm{sat}}-C_{L2}}{C_{\mathrm{sat}}-C_{L1}})=-K_{L}{a(t}_{2}-t_{1}).$$

The dynamical absorption method [27] was applied in order to determine the K_L_a value. This method consists of the elimination of oxygen in the liquid phase to obtain an oxygen concentration close to zero through the injection of nitrogen. This permits to simplify further Equation (7) with t_1_ = 0 and C_L1_ = 0%. Then, the liquid is again put into contact with air and the increase of the dissolved oxygen concentration is measured over the time. The K_L_a can then be deviated through the slope of the $\ln(\frac{C_{\mathrm{sat}}-C_{L2}}{C_{\mathrm{sat}}})$ vs. time plot. Measurements were performed in triplicates.

### 2.11. Mathematical Development of a Growth Model to Describe the Microbial Population Dynamics

The following assumptions are made for the model construction: (i) no oxygen limitation in the biofilm compartment with the packing tower, (ii) the oxygen concentration in the planktonic cell compartment is limited, (iii) the dilution rate is affecting directly the planktonic cell compartment but not the biofilm compartment. The used model parameters are listed in Table 2.

#### 2.11.1. Batch Fermentation

The growth rate of bacteria can be described through the well known Monod equation of growth represented by Equation (8):

(8) $$µ=\frac{r_{x}}{X}=\frac{{µ}_{\max}S}{K_{s}+S}.$$

The total biomass development in the trickle-bed biofilm reactor can be divided into the growth of sessile and planktonic cells. For the planktonic cells, two limiting factors have to be taken into account: the substrate and dissolved oxygen concentration. If oxygen is a limiting factor, the specific growth rate varies with the dissolved oxygen concentration according to the Monod equation like for any other substrate limitation. In our case, oxygen and substrate are complementary substrates and thus, the product rule is applied [28]. The growth speed for the planktonic cells is thus given through Equation (9):

(9) $$r_{x,p}={µ}_{\max}\left(\frac{S}{K_{s}+S}\right)\left(\frac{C_{L}}{K_{o}{+C}_{L}}\right)X_{p}.$$

For the model, Equation (9) was adapted according to the approach used by Roels [29], as shown in Equation (10):

(10) $$r_{x,p}={µ}_{\max}*\min[\left(\frac{S}{K_{s}+S}\right),\left(\frac{C_{L}}{K_{o}{+C}_{L}}\right)]X_{p}.$$

Here, the growth speed of the planktonic cells is assumed to be influenced by the more pronounced limiting factor which means the minimum value of the term representing either the substrate limitation or the limited dissolved oxygen availability.

The biomass development for the planktonic cells can be described by the differential Equation (11):

(11) $$\frac{{\mathrm{dX}}_{p}}{\mathrm{dt}}=r_{x,p}-k_{a}X_{p}+k_{d}X_{b}$$

where k_a_ represents the switching rate from the planktonic state to the sessile state of the cells (adsorption) and k_d_ the releasing rate of the sessile cells to the planktonic state (detachment). Thus, the term k_a_X_p_ correspond to the number of planktonic cells that adhere to the support whereas k_d_X_b_ describes the sessile cells detaching from the support. 

The growth speed for the sessile cells can be described through Equation (12) by taking into account the substrate limitation due to the randomly distributed medium on the packing elements:

(12) $$r_{x,b}={µ}_{\max}\left(\frac{S}{K_{s}+S}\right)X_{b}.$$

In this case, dissolved oxygen limitations are not considered for the sessile cells in the model. It can be assumed that the aeration is very efficient in the packing tower and the biofilm thickness is sufficiently low to neglect oxygen gradients.

The development of the biofilm on the packing elements can be described by Equation (13):

(13) $$\frac{{\mathrm{dX}}_{b}}{\mathrm{dt}}=r_{x,b}+k_{a}X_{p}-k_{d}X_{b}.$$

The terms k_a_X_p_ and k_d_X_b_ represent the corresponding biomass that is adhering or detaching as described above. 

The substrate consumption of the sessile and planktonic cells is given by Equation (14):

(14) $$\frac{\mathrm{dS}}{\mathrm{dt}}=-\frac{r_{x,b}}{Y_{X/S}}-\frac{r_{x,p}}{Y_{X/S}}.$$

The availability of dissolved oxygen can be described by Equation (15):

(15) $$\frac{{\mathrm{dC}}_{L}}{\mathrm{dt}}=k_{L}{a(C}_{\mathrm{sat}}-C_{L})-\frac{r_{x,b}}{Y_{X/O}}-\frac{r_{x,p}}{Y_{X/O}}$$

where the terms $\frac{r_{x,b}}{Y_{X/O}}$ and $\frac{r_{x,p}}{Y_{X/O}}$ represent the oxygen uptake rate of the biofilm and planktonic cells, respectively. For the model, it was assumed that the dissolved oxygen concentrations were equivalent for both compartments due to the continuous recirculation of the medium between the stirred tank reactor and the packing tower.

#### 2.11.2. Continuous Fermentation

For the continuous fermentation, the dilution rate affects only the planktonic phase. The supply and removal of dissolved oxygen through the alimentation and elimination is neglected. This means that Equation (11) describing the development of the planktonic biomass is extended with the term in bold in Equation (16):

(16) $$\frac{{\mathrm{dX}}_{p}}{\mathrm{dt}}=r_{x,p}-k_{a}X_{p}+k_{d}X_{b}-{\mathrm{DX}}_{p}.$$

And Equation (14) describing the substrate consumption is extended as shown by Equation (17):

(17) $$\frac{\mathrm{dS}}{\mathrm{dt}}=-\frac{r_{x,b}}{Y_{X/S}}-\frac{r_{x,p}}{Y_{X/S}}+{D(S}_{\mathrm{in}}-S).$$

The ODEs were coded and solved with Python 3.7 via the Anaconda–Spyder interface using the odeint function (see Data S2 for the code).

## 3. Results

### 3.1. Design of a Two-Compartment Biofilm Reactor to Promote the Biofilm Proliferation

In a previously designed trickle-bed biofilm reactor ([16,25]), the co-existence of a planktonic and biofilm population was recurrently observed during the cultivation of *B. amyloliquefaciens* GA1 which hindered data interpretation and probably decreased the production yield. The actual set-up (Figure 1) is split into two compartments: (i) a stirred bioreactor containing exclusively planktonic cells and (ii) a packing column where the biofilm is attached and on which liquid medium recirculated from the stirred bioreactor is fed. Three constraints have been considered for promoting the proliferation of the biofilm population and to reduce the planktonic one, i.e., a short residence time in the packing column (only the most performant strains will attach) coupled with a high dilution rate through the two-compartment set-up (washing out of planktonic cells) and a strong oxygen limitation in the liquid phase (unfavorable growth conditions in the stirred tank reactor). 

A mean residence time of ~37 s was determined in the packing tower with tracer particles. This is quite short compared to the residence time of ~10 min of the cells in the stirred tank reactor (corresponds to the recirculation time of one reactor volume). Since, in the present case study, the objective was to promote the biofilm formation and decrease the number of planktonic cells, a dilution rate higher than the maximum growth rate of the cells (i.e., D = 0.5 h^−1^) was considered.

In order to avoid foam formation and to limit the growth of planktonic cells in the stirred tank reactor, air was only injected into the compartment containing the packing elements. The oxygen mass transfer to the bulk medium and the planktonic cells occurs only when the liquid phase flows down on the packing tower during recirculation. Whereas the oxygen availability in the stirred tank reactor is strongly limited, the adhered biomass on the structured metal packing benefits from a good gas/liquid mass transfer. 

The volumetric oxygen mass-transfer coefficient K_L_a of the system was determined using a dissolved oxygen probe placed in the stirred tank reactor. The oxygen uptake of the medium occurs only in the packing tower where the air is injected. The structured metal packing elements exhibit a high specific surface area (~500 m^2^m^−3^ [25,30]) and were designed for improving contact between air and liquid phases. The K_L_a measurement was performed without the presence of cells via the dynamical absorption method as described in the Material and Methods Section 2.10. The K_L_a reached a value of 3.0 ± 0.1 h^−1^.

### 3.2. The EPS^+^ Strain Exhibited Enhanced Performance in the Biofilm Reactor

**Figure 3:**
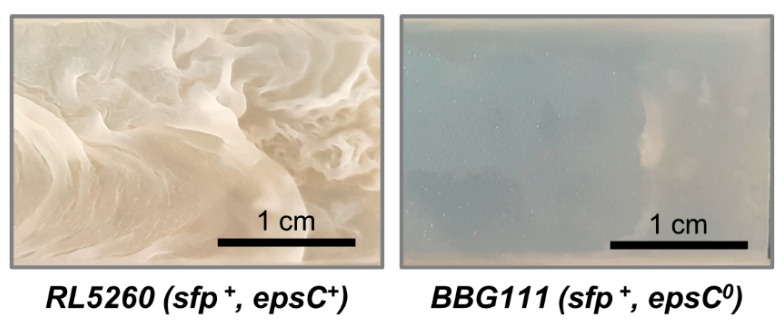
Demonstration of the biofilm development of a strain producing exopolysaccharides (RL5260, left side) or not (BBG111, right side). The images show sections of colonized silicone coupons incubated under identical growth conditions in a drip-flow reactor for 48 h.

Biofilm cultivations with a strong (RL5260) and poor (BBG111) biofilm producing *B. subtilis* 168 strain were performed in the previously described trickle-bed biofilm reactor (cf. Materials and Methods Section 2.3). RL5260 is able to produce exopolysaccharides (EPS), a crucial element for the biofilm matrix formation, whereas BBG111 is deficient in EPS production and thus cell aggregates are formed only in thin layers. Figure 3 demonstrates clearly the different biofilm phenotypes of RL5260 and BBG111 when they were cultivated on silicone coupons in a drip-flow reactor, as described in a previous work [24].

#### 3.2.1. Planktonic Cell Growth and Biofilm Development

**Figure 4:**
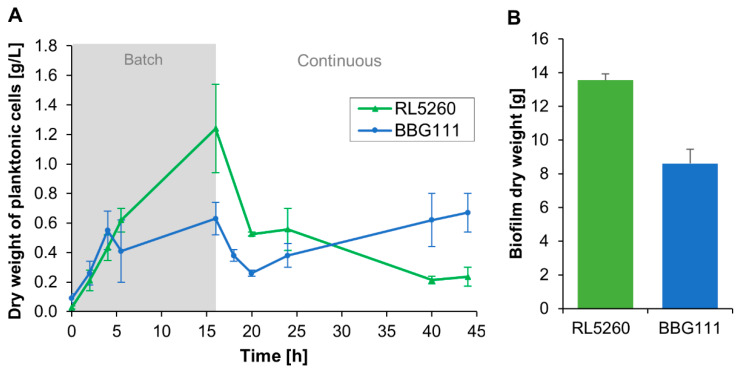
(**A**) Growth curves of planktonic cells and (**B**) amount of adhered dry biomass on the packing tower at the end of the continuous culture measured for each strain in the trickle-bed biofilm reactor. The standard deviation is indicated by error bars.

The growth of the planktonic cells in the trickle-bed biofilm reactor was followed overtime and the weight of the attached biomass on the reactor support has been measured at the end of the cultivation. The results are presented in Figure 4A,B.

During the batch culture (0–16 h), both strains started growing rapidly in the liquid medium which is continuously recirculated between the stirred tank reactor and the packing tower. BBG111 and RL5260 reached a similar maximum specific growth rate of 0.39 ± 0.07 h^−1^ and 0.38 ± 0.04 h^−1^, respectively. After 4 h, the growth of BBG111 remained stagnant and then restarted to increase slightly. After starting the continuous culture, the number of cells in the liquid medium dropped strongly (16–20 h) due to the washing out of cells since the dilution rate (0.5 h^−1^) has been chosen higher than the specific growth rate of the cells in order to eliminate non-adherent cells. During the continuous cultivation phase, the number of planktonic cells decreased for RL5260 whereas for BBG111, the number of planktonic cells increased with the time. Increased standard deviations are probably due to not completely synchronized cultures between the performed repetitions. For the whole cultivation, BBG111 produced 7.8 ± 1.5 g of planktonic cells (dry weight) and RL5260 6.6 ± 1.1 g. BBG111 and RL5260 reached respectively a total amount of 8.6 ± 0.8 and 13.5 ± 0.4 g attached dry biofilm on the packing tower. Hence, RL5260 produced about 1.6 times more adhered biomass than BBG111. This resulted in a biomass ratio of biofilm vs. planktonic cells of 1.2 ± 0.3 for BBG111 and 2.1 ± 0.4 for RL5260. The biomass ratio of RL5260 was 1.8 higher compared to BBG111.

#### 3.2.2. Both Strains Displayed Similar Glucose Consumption Profiles

**Figure 5:**
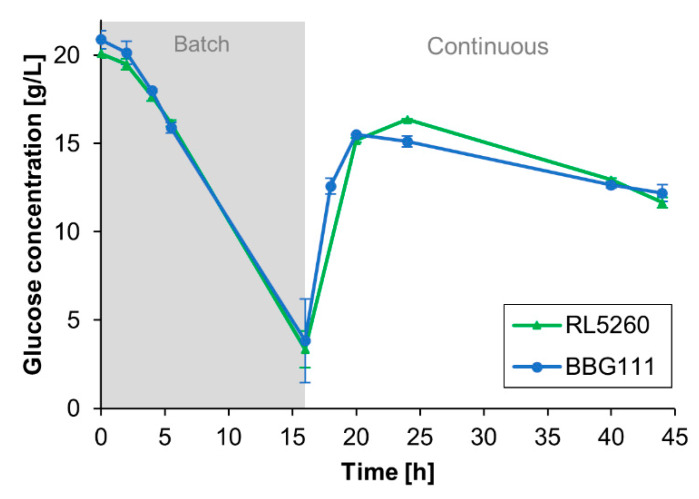
Glucose concentrations present in the bulk medium for the two *B. subtilis* 168 strains during the cultivation. The standard deviation of the measurements is indicated by error bars.

Figure 5 describes the glucose consumption of the strains during the cultivation process. Interestingly, the consumption rates of BBG111 and RL5260 were similar.

For the present system, the substrate-to-biomass conversion yield was 0.16 ± 0.02 g g^−1^ for BBG111 and 0.20 ± 0.01 g g^−1^ for RL5260 calculated for the total biomass produced (planktonic cells and biofilm) per total amount of consumed glucose. For comparison, a substrate-to-biomass conversion yield Y_X/S_ of 0.22 ± 0.02 g g^−1^ for BBG111 and 0.26 ± 0.03 g g^−1^ for RL5260 was determined from shake flasks experiments during the exponential growth phase by measuring the glucose consumption and the corresponding cell dry weight. 

#### 3.2.3. Increased Biofilm Development Enhanced the Surfactin Productivity 

The mean surfactin productivities determined for both stains are summarized in Table 3. The mean surfactin productivity was comparable for both strains during the initial batch cultivation step. Yet, the mean surfactin productivity of the strong biofilm former RL5260 was about 37% higher during the continuous phase compared to the mean productivity of BBG111.

#### 3.2.4. Carbon Utilization Pointed out a Totally Different Biofilm Formation Rate between the Two *B. subtilis* Strains

**Figure 6:**
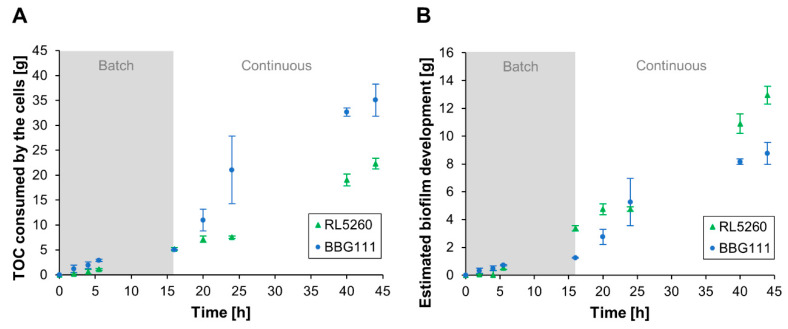
(**A**) Cumulative total organic carbon (TOC) consumption by the cells during the cultivation of the two *B. subtilis* 168 strains. (**B**) Cumulative biofilm development on the packing elements estimated via the TOC mass balance and obtained biomass-TOC conversion yields. Error bars indicate the standard deviation of the measurements.

The overall glucose consumption in the system did not show any difference between the two *B. subtilis* strains although the biofilm development and surfactin production was significantly increased for RL5260. In order to examine the carbon consumption by the cells, a TOC mass balance was performed for elucidating the behavior of the different strains. Figure 6A shows the results of the TOC analysis for both strains. 

The estimated TOC consumption of the cells was similar for both stains during the batch cultivation phase. Surprisingly, BBG111 showed a significant increased TOC consumption during the continuous cultivation compared to RL5260, although BBG111 produced a significant lesser amount of adhered biomass than RL5260. During the continuous cultivation phase, the TOC consumption can be mainly attributed to the biofilm. The TOC consumption of the planktonic cells can be neglected since they are largely washed out due to the high dilution rate or mainly derived from the biofilm as a consequence of detachment. Thus, the results demonstrate different metabolic behaviors between RL5260 and BBG111 during biofilm formation.

As a result, the yield of the produced biofilm per consumed TOC (Y_biofilm/TOC_) was found to be more than two times higher for RL5260 (0.61 ± 0.05 g g^−1^) than for BBG111 (0.25 ± 0.05 g g^−1^). Based on these conversion yields, we were able to plot a biofilm development curve during the cultivation, as presented in Figure 6B. At the end of the batch phase (at 16 h), RL5260 developed a significantly higher amount of adhered biomass. After the start of the continuous phase, the biofilm development of RL5260 seemed to be reduced and even to be stagnant around 20 h according to the TOC measurements. Probably, RL5260 took some time to adapt to the high dilution rate. After the adaptation, the growth of RL5260 restarted strongly until the end of the cultivation. BBG111 appeared to develop an increased adhered biomass upon starting the continuous phase but then the growth slowed down, probably as a result of cell detachment due to limited adhesion capacities. In order to verify the estimated biofilm development via the TOC measurements, a bacterial growth model was developed to predict the biofilm development kinetics in the two-compartment system as presented in the following section.

### 3.3. Modeling of Microbial Population Dynamics

Two subpopulations of cells are co-existing in the biofilm bioreactor, i.e., the planktonic cells mainly present in the stirred tank reactor and the sessile cells adhered to the packing tower. The growth dynamics of the planktonic cells could be measured during the cultivation experiment. However, it was challenging to get more information about the growth dynamic of the biofilm on the packing tower. An established TOC mass balance (see Section 3.2.4) led to more information about the biofilm development on the packing elements for both strains. However, the reliability of the biomass-TOC conversion yield was not certainly approved. Hence, we developed a microbial growth model based on ODEs for predicting the growth behavior of the biofilm in the system to get a deeper insight into the populations’ behavior. The model was fed with some parameters measured in this work and with appropriate parameters described in literature that are listed in Table 4. The outcome of the model was then compared to the measured values for verification and validation. 

The substrate affinity constant K_s_ was set to 0.015 g L^−1^ as used by Guez et al. [31] in a previous work in our laboratory for modelling fed-batch cultures of *B. subtilis* in Landy medium.

For the dissolved oxygen saturation concentration, C_sat_ = 6.73 mg L^−1^ was used. The value corresponds to the oxygen concentration at saturation in water at 37 °C and has been extracted from the online data base DOTABLES (https://water.usgs.gov/software/DOTABLES/). For the oxygen-biomass conversion coefficient Y_X/O_, a value of 1 g g^−1^ was given as used by Lin et al. [32] and Xu et al. [33]. The oxygen affinity constant K_o_ was set to 0.001 g L^−1^, a mean value of the affinity constants found by Guisasola et al. [34].

For the oxygen mass transfer, an estimated correction factor for the determined K_L_a value was introduced given that the oxygen mass transfer was only determined with the medium in the absence of biomass. The presence of microorganism affects significantly the oxygen mass transfer rate as a result of cell respiration [35]. The phenomenon that the oxygen uptake rate (OUR) increases with the cell concentration coupled to an increase in K_L_a is called biologically enhanced oxygen transfer [36] and can be characterized by an enhancement factor E [27]. In the literature, enhancement factors up to 5 are described in the presence of high cell concentrations [36]. In the present system, an additional high interfacial oxygen transfer occurs through the direct contact of adhered cells with the injected air resulting in an increased total oxygen transfer [26]. Surfactin production in *B. subtilis* depends strongly on the oxygenation. For an appropriate surfactin production a K_L_a value over 10.8 h^−1^ is necessary, as shown by Fahim et al. [37]. Comparable surfactin production rates to this work were achieved by Yeh et al. [38] in a foaming bioreactor with solid carriers with a K_L_a value of 30.96 h^−1^ using *B. subtilis* ATC 21332 and Coutte et al. [15] in a bubbleless membrane bioreactor with a K_L_a value of 40 h^−1^ by using a *B. subtilis* 168 derivative strain. K_L_a values between 10 and 40 were tested on the model, the most appropriate value was 24 resulting in an enhancement factor of 8.

Two hypotheses were verified with the established model:

(i) The significant difference in biofilm development of RL5260 and BBG111 was due to unequal adhesion capacities as a result of the presence or not of EPS.
(ii) The high dilution rate during the continuous fermentation exerted a strong washing out of the planktonic cells. No additional cell adhesion occurred on the packing elements, only cell detachment took place.

**Figure 7:**
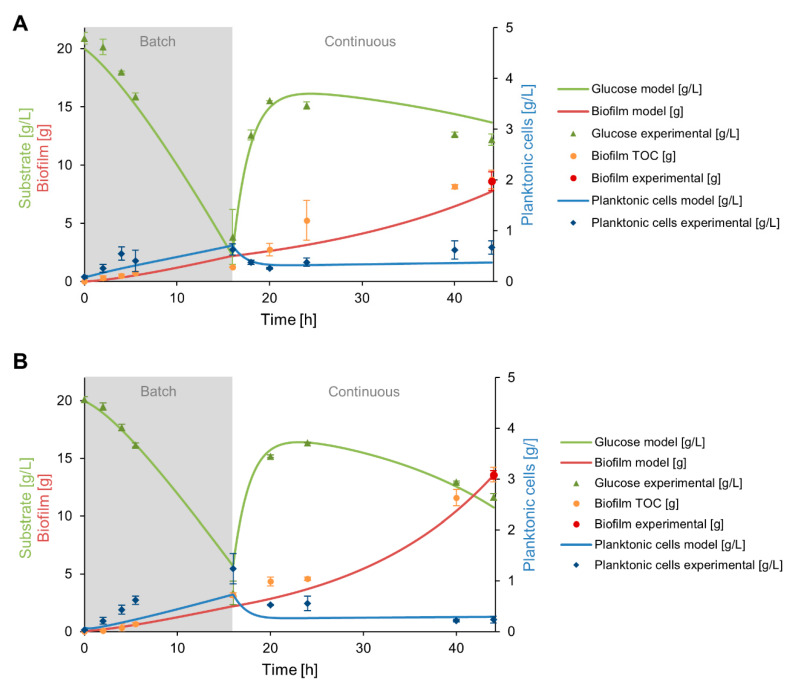
Glucose consumption, planktonic cells and biofilm development predicted by the model for the two-compartment system for BBG111 (**A**) and RL5260 (**B**). The model results are drawn with a continuous line and the experimental results are added as point references for comparison.

For testing the first hypothesis, the different adhesion capacities of BBG111 and RL5260 were modelized through different k_a_ and k_d_ values and thus, a different k_a_/k_d_ ratio during the batch cultivation phase. Though, the values were orientated on the previously mentioned biofilm vs. planktonic cells ratio for both strains (Section 3.2.1). For the second hypothesis k_a_ was set to zero in the model for the continuous cultivation phase. Table 5 summarizes the introduced parameters. The model results and the corresponding experimental results are presented in Figure 7A for BBG111 and in Figure 7B for RL5260.

The simulations show that the established growth model is able to describe the development of the biofilm and planktonic population in the two-compartment reactor. The model predicted properly the development of the planktonic cells for BBG111 (Figure 7A) when compared to the experimental values. For the biofilm development, the model represented well the experimental values at the beginning of the batch phase and during the continuous phase. However, the values at the end of the batch phase was slightly overestimated. The glucose consumption was quite correctly predicted during the batch cultivation and for the continuous cultivation until 20 h. After that time point, the prediction and experimental values are diverging which resulted in a light underestimation of the consumed glucose. 

For RL5260 (Figure 7B), the model predicted less accurate the development of the planktonic phase during batch cultivation regarding the experimental values. However, the biofilm development seemed to fit with the measured biofilm development via the TOC analysis and the final biofilm dry weight value measured on the packing elements. The glucose consumption was correctly predicted compared to the experimental values, except for the time point t = 16 h where the model predicted slightly higher values than measured in the system. 

The overall model predictions were close to the experimental values. A Chi-square goodness of fit test confirmed that there were no significant differences between the observed and predicted values for both strains with a significance level of α = 0.05. All calculated p-values were extremely high, which resulted in the acceptance of the null hypothesis that no significant differences exist between the observed and predicted values (see Data S3 for the test results, Table S1 for BBG111 and Table S2 for RL5260).

## 4. Discussion

The objective of this work was to develop a model able to describe the growth dynamics of the biofilm and planktonic population present in the designed trickle-bed biofilm reactor in order to understand better the behavior of the system for further process intensification. In particular, biofilm development on the packing elements gives important information about the process, but is difficult to monitor during cultivation. The growth model was used in order to confirm the two hypotheses that the significant difference in biofilm development of BBG111 and RL5260 is linked to the production or not of EPS, and that the high dilution rate washes out the non-adherent or detaching cells in the designed system.

The experimental data are in good accordance with those obtained with the developed growth model by using a combination of the first hypothesis (different k_a_/k_d_ ratio during batch cultivation) and the second one (k_a_ = 0 during continuous cultivation) for both strains. This was confirmed by a Chi-square goodness to fit test with a confidence level of α = 0.05. The two hypotheses made initially for the present system have thus been validated. The model also confirmed the biofilm development dynamics determined via experimental TOC measurements and the established TOC mass balance. 

The increased k_a_/k_d_ ratio for RL5260 during the batch cultivation was linked to the capacity of EPS secretion which has shown to improve the colonization capacity and reduce cell detachment. The presence of EPS permitted RL5260 to build up a functional biofilm structure and to protect the adhered cells from external influences. Once adhered, the cells produced EPS and proliferated on the packing elements to construct their own environment. Several works on *B. subtilis* biofilm formation have shown that EPS production facilitates cell spreading and promotes the colonization of a solid support [24,39,40]. Since BBG111 is a poor biofilm former and does not produce EPS, the biofilm formation capacities were reduced (lower k_a_/k_d_ ratio) and cell detachment occurred more frequently after the cell adhesion step than in the case of RL5260. Moreover, the additional high dilution rate carried out a strong selective pressure on the planktonic cells and limited the re-adherence during the continuous cultivation due to the washing out of the planktonic cells (k_a_ = 0).

Globally, BBG111 and RL2560 produced comparable amounts of planktonic cells. RL5260 produced more planktonic cells during the batch phase. However, when the continuous cultivation phase was launched, the planktonic cells were mostly washed out for RL5260 whereas the number of planktonic cells of BBG111 increased during the continuous phase. This was probably a result of the limited adhesion capacities of this strain due to the absence of EPS. The maximum specific growth rates of 0.39 h^−1^ and 0.38 h^−1^ for BBG111 and RL5260 were comparable in the two-compartment system. They were close to the values of 0.35 h^−1^ and 0.38 h^−1^ determined by Guez et al. [31] and Martínez et al. [41] as growth rates for *B. subtilis* in glucose-limited fed-batch cultures. However, the EPS^+^ strain RL5260 produced about 1.6 more adhered biomass than BBG111 (EPS^-^) which resulted in an important difference regarding the biofilm versus planktonic cell ratio. This ratio was nearly two times higher for RL5260. 

Although EPS production is advantageous for cell adhesion and leads to enhanced biofilm formation, it is metabolically expensive [42]. Thus, EPS production may reduce the cell growth and affects negatively the surfactin production. Nevertheless, the results have shown that the mean surfactin productivity of the strain RL5260 with increased biofilm formation capacity through EPS production was about 37% improved during the continuous phase compared to BBG111. This indicates clearly the improved performance of the EPS^+^ strain in this system compared to the EPS^-^ strain. 

Surprisingly, both strains showed a similar glucose consumption profile when the concentration was measured in the bulk medium. For the same amount of consumed glucose, RL5260 produced significantly more adhered biomass as well as higher amounts of surfactin than BBG111. This indicated that both strains had a completely different cell physiology in the system due to the differences in EPS production. 

Regarding the performed TOC measurements, the TOC consumption profile for BBG111 was significantly increased compared to RL5260 during the continuous phase. This was most likely linked to the different biofilm development capacities due to the production of EPS or not of RL5260 and BBG111. Hence, RL5260 and BBG111 used the available carbon source in the medium in a different way. Given that BBG111 is not able to synthesize a biofilm matrix, the adhered biomass consisted mainly of cells whereas the adhered biomass of RL5260 contained a mixture of cells and biofilm matrix. Biofilm composition measurements of RL5260 that were performed in our laboratory using biofilms developed on drip-flow reactor coupons revealed a relative EPS amount of 81% and a cell content of 19%. Both strains show comparable glucose-to-biomass conversion yields for the cellular production in suspended cell cultures (Y_X/S,cells_ of 0.22 g g^−1^ for BBG111 and Y_x/s_,_cells_ = 0.26 g g^−1^ for RL5260). The yields were similar or close to the yield of 0.22 g g^−1^ previously reported by Guez et al. [31] for *B. subtilis* ATCC6633 grown in Landy medium in shaking flasks. Assuming a substrate-to-EPS conversion yield that is significantly higher than the conversion yield for cellular production, e.g., Y_x/s,EPS_ ~ 0.57 g g^−1^ as obtained by Huang et al. for the production of poly-γ-glutamic acid (PGA), a major extracellular compound of *B. subtilis* CGMCC1250 [43], RL5260 used in total lesser amounts of carbon sources than BBG111 for the biofilm development. This assumption is further confirmed through the determined yield of the produced biofilm per consumed TOC Y_biofilm/TOC_ for both strains. RL5260 reached a yield of Y_biofilm/TOC_ = 0.61 g g^−1^ whereas BBG111 reached only 0.25 g g^−1^. This shows the lower energy consumption of RL5260 for the biomass production due to the increased biosynthesis of EPS instead of cells. 

The reduced energy consumption of RL5260 for the biofilm development resulted in a more efficient surfactin production. It can be considered that surfactin was mainly produced by the cells present in the biofilm since a sufficient aeration is necessary for the production which was not guaranteed for the planktonic cells in the stirred tank reactor. Consequently, the specific surfactin production was significantly increased for RL5260. Hence, RL5260 reached a mean specific surfactin production of 90 mg L^−1^ h^−1^ per g of adhered cell dry weight whereas BBG111 produced only 20 mg L^−1^ h^−1^ per g of adhered cell dry weight. 

In conclusion, the two-compartment biofilm reactor designed in this study has shown to be suitable for continuous surfactin production. The EPS^+^ strain exhibited significantly improved performances in terms of cell adhesion and surfactin production in this system by comparison with the EPS^−^ strain. The surfactin yield and population stability inside the reactor could be further improved by engineering the biofilm formation capacity of the cells. For a good process performance, a trade-off between enhanced cell adhesion and increased productivity has to be chosen. EPS production could be modulated in favor of surfactin production by guaranteeing a sufficient cell adhesion through a controlled EPS production while increasing the numbers of potential cell factories. Moreover, cell morphology engineering could improve cell adhesion and further reduce cell detachment. 

## Figures and Tables

**Table 1:** Strains used in this study.

*B. subtilis* Strains	Genotype	Source
**BBG111**	*trpC2*, *sfp*^+^, *epsC*^0^; *Cm^R^*	[22]
**RL5260**	*trpC2*, *sfp*^+^, *epsC*^+^; *Erm^R^*	[23]

**Table 2:** List of parameters used for the model construction.

Parameter	Description	Unit
**µ_max_**	Maximum growth rate of cells	h^−1^
**C_L_**	Dissolved oxygen concentration	g L^−1^
**C_sat_**	Dissolved oxygen concentration at saturation	g L^−1^
**k_a_**	Switching rate liquid to biofilm (adsorption)	h^−1^
**k_d_**	Switching rate biofilm to liquid phase (detachment)	h^−1^
**K_L_a**	Volumetric oxygen mass-transfer coefficient	h^−1^
**K_o_**	Oxygen affinity constant	g L^−1^
**K_s_**	Substrate affinity constant	g L^−1^
**r_x,b_**	Growth speed sessile cells	g L^−1^ h^−1^
**r_x,p_**	Growth speed planktonic cells	g L^−1^ h^−1^
**S**	Substrate concentration in the reactor	g L^−1^
**S_in_**	Substrate concentration at the reactor entry	g L^−1^
**X_b_**	Biofilm biomass concentration	g L^−1^
**X_p_**	Planktonic biomass concentration	g L^−1^
**Y_X/O_**	Oxygen-biomass conversion coefficient	g g^−1^
**Y_X/S_**	Substrate-biomass conversion coefficient	g g^−1^

**Table 3:** Surfactin productivity of the two *B. subtilis* 168 strains measured in the bulk medium during batch and continuous cultivation.

Cultivation Phase		*BBG111* (*sfp*^+^, *epsC*^0^)	*RL5260* (*sfp*^+^, *epsC*^+^)
**Batch**	Mean surfactin productivity (mg L^−1^ h^−1^)	107.4 ± 5.6	130.4 ± 25.3
**Continuous**	Mean surfactin productivity (mg L^−1^ h^−1^)	168.1 ± 22.0	231.0 ± 14.2

**Table 4:** General model parameters and their corresponding values used for *B. subtilis* BBG111 (*sfp*^+^, *epsC*^0^) and RL5260 (*sfp*^+^, *epsC*^+^).

Parameter	Description	Unit	BBG111	RL5260
**µ_max_**	Max. growth rate of cells	h^−1^	0.39	0.38
**C_sat_**	Dissolved oxygen concentration at saturation	g L^−1^	0.00673	0.00673
**E**	Biological enhancement factor for K_L_a	-	8	8
**K_L_a**	Volumetric oxygen mass-transfer coefficient	h^−1^	3	3
**K_o_**	Oxygen affinity constant	g L^−1^	0.001	0.001
**K_s_**	Substrate affinity constant	g L^−1^	0.015	0.015
**S_in_**	Substrate concentration at the reactor entry	g L^−1^	20.00	20.00
**Y_X/O_**	Oxygen-biomass conversion coefficient	g g^−1^	1.00	1.00
**Y_X/S_**	Substrate-biomass conversion coefficient	g g^−1^	0.16	0.20

**Table 5:** Parameters related to cell adhesion and detachment that were introduced into the model.

Parameter	Description	Unit	BBG111	RL5260
**k_a_**	Switching rate to biofilm (adsorption) (batch/continuous)	h^−1^	(0.6/0)	(2.1/0)
**k_d_**	Releasing rate to planktonic state (detachment) (batch/continuous)	h^−1^	(0.5/0.345)	(1/0.315)
**k_a_/k_d_**	Ratio switching / releasing rate (batch/continuous)	-	(1.2/-)	(2.1/-)

## Supplementary Materials

The following are available online at https://www.mdpi.com/2076-2607/8/5/679/s1, S1: Determination of the biofilm dry weight percentage, S2: Python 3.7 code of the established growth model, S3: Statistical analysis: Chi-square goodness of fit test; Table S1. Results of the Chi-square goodness of fit test for the model and experimental data obtained with BBG111; Table S2. Results of the Chi-square goodness of fit test for the model and experimental data obtained with RL5260.
