# Supplementary material for: Growth Dynamics of Bacterial Populations in a Two-Compartment Biofilm Bioreactor Designed for Continuous Surfactin Biosynthesis
Source: Microorganisms. 2020 May 7;8(5):679. doi: 10.3390/microorganisms8050679 (PMC7285194; doi:10.3390/microorganisms8050679)
Supplement: Supplementary file 1 [file microorganisms-08-00679-s001.pdf]

Supplementary data

Growth Dynamics of Bacterial Populations in a Two-Compartment Biofilm Bioreactor Designed for Continuous Surfactin Biosynthesis

Hannah Luise Brück [1,2], François Coutte [2], Pascal Dhulster [2], Sébastien Gofflot [3], Philippe Jacques [1] and Frank Delvigne [1,*]

[1] MiPI, TERRA Teaching and Research Centre, Joint Research Unit BioEcoAgro N° 1158, Gembloux Agro-Bio Tech, University Liège, University Lille, INRAE, UPJV, YNCREA, University Artois, University Littoral Côte d'Opale, B-5030 Gembloux, Belgium, hannah.bruck@doct.uliege.be (H.L.B.); philippe.jacques@uliege.be (P.J.)

[2] ICV – Institut Charles Viollette, Joint Research Unit BioEcoAgro N° 1158, University Lille, INRAE, University Liège, UPJV, YNCREA, University Artois, University Littoral Côte d'Opale, F-59000 Lille, France, francois.coutte@polytech-lille.fr (F.C.); pascal.dhulster@univ-lille.fr (P.D.)

[3] Walloon Agricultural Research Center (CRA-W), Agricultural Product Technology Unit, Chaussée de Namur, 24, B-5030 Gembloux, Belgium, s.gofflot@cra.wallonie.be (S.G.)

* Correspondence: f.delvigne@uliege.be (F.D.)

S1 Determination of the biofilm dry weight percentage

It was not possible to dry the packing elements because the biomass is extremely difficult to remove once dried and this operation would spoil the packing elements for the next use. Therefore, a conversion factor from wet to dry biomass has been determined using cell cultures in flasks containing a small packing element of the same material. The flasks were incubated at 37°C with a low rotation rate (100 rpm) allowing the formation of a biofilm on the packing element. After 72h, the packing element was weighted to get the wet biofilm weight and then dried in the oven at 105°C during 48h to measure subsequently the dry biofilm weight. The cultures were performed in triplicates. A biofilm dry weight percentage of 7.9 ± 0.6 % for RL5260 and 7.5 ± 0.7 % for BBG111 could be determined.

S2 Python 3.7 code of the established growth model

```
# -*- coding: utf-8 -*-

import numpy as np
from matplotlib import pyplot as plt
from scipy.integrate import odeint
import pandas as pd
import xlsxwriter

#Create the function for the batch phase and define the variables
def batch(state,t):
  X_p = state[0]
  X_b = state[1]
  S = state[2]
  C_L = state[3]
  mu = 0.38 # growth rate (h-1)
  Ks =  0.015 #substrate affinity constant (g/L)
  Yxs = 0.2 #conversion coefficient (g/g)
  Yxo = 1 #conversion coefficient (g/g)
  Kla = 24 #oxygen transfer rate (h-1)
  Ko = 0.001 #affinity constant (g/L)
  ka = 2.1 #switching rate to biofilm (h-1)
  C_sat = 0.00673 # saturation for dissolved oxygen (g/L)
  kd = 1 #releasing rate to the planktonic phase (h-1)


  rx_p = mu * min (S/Ks+S , C_L/Ko+C_L)*X_p # Roels approach
  rx_b = mu*(S/(Ks+S))*X_b
  dX_pdt = rx_p - ka*X_p + kd*X_b
  dX_bdt = rx_b + ka*X_p - kd*X_b
  dSdt = -rx_p/Yxs -rx_b/Yxs
  dC_Ldt = Kla*(C_sat-C_L)-(rx_p/Yxo)-(rx_b/Yxo)

  return [dX_pdt,dX_bdt,dSdt,dC_Ldt]

#Define initial conditions and call odeint to generate solution
t = np.arange(0,16,0.1)
state0 = [0.08,0,20,0.00673]
state = odeint(batch,state0,t)
```

```
    Pfinal= state[159,0]
    Bfinal = state[159,1]
    Sfinal = state[159,2]
    C_Lfinal = state [159,3]

    #Create and plot figure
    plt.figure(1)
    plt.plot(t,state[:,0], 'g')
    plt.plot(t,state[:,1], 'b')
    plt.plot(t,state[:,2], 'k')

    #Create a data frame
    data1=(state[:,0])
    data2=(state[:,1])
    data3=( state[:,2])
    dataframe=pd.DataFrame(
            {'Time': t,
             'Planktonic cells':data1,
             'Biofilm': data2,
             'Substrate': data3})
    writer_object      =      pd.ExcelWriter('RL5260_Cultivation_Batch.xlsx',
engine='xlsxwriter')
    dataframe.to_excel(writer_object, sheet_name='Batch',
                       startrow=1)

    #Create xlsxwriter workbook object
    workbook_object = writer_object.book

    #Create xlsxwriter worksheet object
    worksheet_object = writer_object.sheets['Batch']

    #Close the Pandas Excel writer
    #Object and output the Excel file.
    writer_object.save()


    #Create the function for the continuous phase and define the variables
    def continuous(state,t):
      X_p = state[0]
      X_b = state[1]
      S = state[2]
      C_L = state[3]
      mu = 0.38 # growth rate (h-1)
      Ks =  0.015 #substrate affintiy constant (g/L)
      Yxs = 0.2 #conversion coefficient (g/g)
      Yxo = 1 #conversion coefficient (g/g)
      Kla = 24 #oxygen transfer rate (h-1)
      Ko = 0.001 #affinity constant (g/L)
      ka = 0 #switching rate to biofilm (h-1)
      C_sat = 0.00673 # saturation for dissolved oxygen (g/L)
      kd = 0.315 #releasing rate to the planktonic phase (h-1)
      D = 0.5 # dilution rate (h-1)
      Sin = 20 #glucose (g/L)
```

```
        rx_p = mu * min (S/Ks+S , C_L/Ko+C_L)*X_p-D*X_p
        rx_b = mu*X_b

        dX_pdt = rx_p - ka*X_p + kd*X_b - D*X_p
        dX_bdt = rx_b + ka*X_p - kd*X_b
        dSdt = -rx_p/Yxs -rx_b/Yxs+D*(Sin-S)
        dC_Ldt = Kla*(C_sat-C_L)-rx_p/Yxo-rx_b/Yxo

        return [dX_pdt,dX_bdt,dSdt,dC_Ldt]

    t = np.arange(16,44.1,0.1)

    #Define initial conditions and odeint to generate the solution
    state0 = [Pfinal,Bfinal,Sfinal, C_Lfinal]
    state = odeint(continuous,state0,t)

    #Add data to figure (1)
    plt.figure(1)
    plt.plot(t,state[:,0], 'g', label='Planktonic cells')
    plt.plot(t,state[:,1], 'b', label='Biofilm')
    plt.plot(t,state[:,2], 'k', label='Substrate')

    plt.ylim([0,20])
    plt.xlabel('Time (h)')
    plt.ylabel('Glucose (g/L), planktonic cells (g/L), biofilm (g)')
    plt.legend(bbox_to_anchor=(1.05,1), loc=2, borderaxespad=0.)
    plt.title('Cultivation')

    Pfinal_con= state[280,0]
    Bfinal_con = state[280,1]
    Sfinal_con = state[280,2]
    C_Lfinal_con = state [280,3]

    #Create a data frame
    data1=(state[:,0])
    data2=(state[:,1])
    data3=( state[:,2])
    dataframe=pd.DataFrame(
            {'Time': t,
             'Planktonic cells':data1,
             'Biofilm': data2,
             'Substrate': data3})
    writer_object   =   pd.ExcelWriter('RL5260_Cultivation_Continuous.xlsx',
engine='xlsxwriter')
    dataframe.to_excel(writer_object, sheet_name='Continuous',
                       startrow=1)

    #Create xlsxwriter workbook object
    workbook_object = writer_object.book

    #Create xlsxwriter worksheet object
    worksheet_object = writer_object.sheets['Continuous']

    #Close the Pandas Excel writer
```

```
#Object and output the Excel file
writer_object.save()
```

S3 Statistical analysis: Chi-square goodness of fit test

The following null hypothesis H_0 has been defined: no significant differences exist between the observed and predicted values with a significance level of α = 0.05. The results of the Chi-square goodness of fit test are presented in **Table S 1** for BBG111 and in **Table S 2** for RL5260.

Table S 1. Results of the Chi-square goodness of fit test for the model and experimental data obtained with BBG111.

	BBG111										
	Glucose [g/L]				Planktonic cells [g/L]				Biofilm dry weight [g]		
#	Observed (mean value)	Estimated	Chi-sq	#	Observed (mean calue)	Estimated	Chi-sq	#	Observed (mean value)	Estimated	Chi-sq
1	20.89	20.00	0.0396	1	0.09	0.08	0.0012	1	0.00	0.00	0.0000
2	20.15	18.54	0.1398	2	0.26	0.17	0.0420	2	0.30	0.14	0.1907
3	17.99	16.71	0.0985	3	0.55	0.26	0.3275	3	0.48	0.35	0.0520
4	15.89	15.18	0.0332	4	0.41	0.32	0.0271	4	0.71	0.53	0.0603
5	3.84	2.37	0.9124	5	0.63	0.71	0.0084	5	1.25	2.19	0.4079
6	15.52	14.93	0.0229	6	0.26	0.33	0.0155	6	2.74	2.63	0.0051
7	15.12	16.13	0.0638	7	0.38	0.32	0.0096	7	5.26	3.15	1.4153
8	12.66	14.38	0.2067	8	0.62	0.36	0.1783	8	8.15	6.46	0.4405
9	12.19	13.66	0.1591	9	0.67	0.37	0.2323	9	8.76	7.74	0.1343

DF	Sum Chi-Sq	p-value		DF	Sum Chi-Sq	p-value		DF	Sum Chi-Sq	p-value
8	1.6759	0.9894		8	0.8420	0.9991		7	2.7061	0.9108

Table S 2. Results of the Chi-square goodness of fit test for the model and experimental data obtained with RL5260.

	RL5260										
	Glucose [g/L]				Planktonic cells [g/L]				Biofilm dry weight [g]		
#	Observed (mean value)	Estimated	Chi-Sq	#	Observed (mean value)	Estimated	Chi-Sq	#	Observed (mean value)	Estimated	Chi-Sq
1	20.10	20.00	0.0005	1	0.03	0.08	0.0324	1	0.00	0.00	0.0000
2	19.49	18.93	0.0167	2	0.21	0.10	0.1195	2	0.08	0.19	0.0669
3	17.68	17.47	0.0026	3	0.43	0.18	0.3665	3	0.33	0.41	0.0148
4	16.15	16.21	0.0002	4	0.62	0.24	0.6022	4	0.66	0.60	0.0059
5	3.35	5.78	1.0187	5	1.24	0.73	0.3605	5	3.17	2.20	0.4317
6	15.19	15.73	0.0184	6	0.53	0.27	0.2300	6	4.35	2.85	0.7870
7	16.36	16.37	0.0000	7	0.56	0.27	0.3174	7	4.58	3.69	0.2120
8	12.94	12.55	0.0120	8	0.21	0.29	0.0187	8	11.59	10.45	0.1248
9	11.66	10.75	0.0774	9	0.24	0.29	0.0104	9	13.59	13.56	0.0001

DF	Sum Chi-Sq	p-value		DF	Sum Chi-Sq	p-value		DF	Sum Chi-Sq	p-value
8	1.1466	0.9971		8	2.0576	0.9792		7	1.6432	0.9769

The null hypothesis is not rejected, all p-values are much higher than 0.05. This means no significant differences exist between the observed and predicted values for both strains.
